# Homeostatic regulation of sleep in the white-crowned sparrow (*Zonotrichia leucophrys gambelii*)

**DOI:** 10.1186/1471-2202-9-47

**Published:** 2008-05-27

**Authors:** Stephany G Jones, Vladyslav V Vyazovskiy, Chiara Cirelli, Giulio Tononi, Ruth M Benca

**Affiliations:** 1 Neuroscience Training Program, University of Wisconsin-Madison, USA; 2 Department of Psychiatry, University of Wisconsin, 6001 Research Park Blvd, Madison, WI 53719, USA

## Abstract

**Background:**

Sleep is regulated by both a circadian and a homeostatic process. The homeostatic process reflects the duration of prior wakefulness: the longer one stays awake, the longer and/or more intense is subsequent sleep. In mammals, the best marker of the homeostatic sleep drive is slow wave activity (SWA), the electroencephalographic (EEG) power spectrum in the 0.5–4 Hz frequency range during non-rapid eye movement (NREM) sleep. In mammals, NREM sleep SWA is high at sleep onset, when sleep pressure is high, and decreases progressively to reach low levels in late sleep. Moreover, SWA increases further with sleep deprivation, when sleep also becomes less fragmented (the duration of sleep episodes increases, and the number of brief awakenings decreases). Although avian and mammalian sleep share several features, the evidence of a clear homeostatic response to sleep loss has been conflicting in the few avian species studied so far. The aim of the current study was therefore to ascertain whether established markers of sleep homeostasis in mammals are also present in the white-crowned sparrow (*Zonotrichia leucophrys gambelii*), a migratory songbird of the order Passeriformes. To accomplish this goal, we investigated amount of sleep, sleep time course, and measures of sleep intensity in 6 birds during baseline sleep and during recovery sleep following 6 hours of sleep deprivation.

**Results:**

Continuous (24 hours) EEG and video recordings were used to measure baseline sleep and recovery sleep following short-term sleep deprivation. Sleep stages were scored visually based on 4-sec epochs. EEG power spectra (0.5–25 Hz) were calculated on consecutive 4-sec epochs. Four vigilance states were reliably distinguished based on behavior, visual inspection of the EEG, and spectral EEG analysis: Wakefulness (W), Drowsiness (D), slow wave sleep (SWS) and rapid-eye movement (REM) sleep. During baseline, SWA during D, SWS, and NREM sleep (defined as D and SWS combined) was highest at the beginning of the major sleep period and declined thereafter. Moreover, peak SWA in both SWS and NREM sleep increased significantly immediately following sleep deprivation relative to baseline.

**Conclusion:**

As in mammals, sleep deprivation in the white-crowned sparrow increases the intensity of sleep as measured by SWA.

## Background

Sleep is regulated by both a circadian and a homeostatic process. The circadian process is the reflection of an endogenously generated rhythm that serves to constrain sleep to the appropriate time throughout the 24-hour period. The homeostatic process is a reflection of the duration of prior wakefulness: extending the duration of wakefulness results in a compensatory increase in the intensity and/or duration of subsequent sleep, suggesting that sleep has some important, and yet unknown, functions [1]. A vast amount of data indicates that the electroencephalographic (EEG) activity in the low frequency range (0.5–4 Hz) of non-rapid eye movement (NREM) sleep, referred to as slow wave activity (SWA, or delta activity), is currently the best marker of the homeostatic sleep drive in mammals. NREM SWA (from now on SWA) declines during normal sleep, with SWA lowest toward the end of a sleep period when sleep need is minimal. Sleep deprivation results in increases in SWA during recovery sleep relative to normal physiological sleep; this phenomenon has been demonstrated in a variety of mammalian species including humans, squirrels, rats, mice and hamsters [2-8]. Further evidence for SWA as a marker of sleep need and intensity is provided by the fact that SWA increases in a regional, use-dependent manner; those brain regions that undergo the greatest waking-associated activity also show the highest regional increases in SWA during subsequent sleep [9-14]. Recent data also suggests that SWA increases specifically in those brain regions that during waking have presumably undergone synaptic potentiation. For instance, SWA increases locally after a learning task and is positively correlated with post-sleep performance improvement [15], suggesting that SWA may represent more than a measure of sleep need and intensity, but may in fact mediate the restorative function of NREM sleep [15-17].

Avian and mammalian sleep share several important features. Notably, birds are the only non-mammalian class to show both rapid eye movement (REM) and NREM sleep [18]. Although little data are available on sleep regulatory mechanisms in birds, the few published studies suggest that both the neural and neurochemical control of REM and NREM sleep likely overlap between the two classes [19,20]. Moreover, as in mammals, sleep amounts in birds are higher during early developmental periods, and sleep quality and quantity decline with age [21,22]. Furthermore, sleep loss has shown to negatively impact performance and vigilance in both birds and mammals, whereas sleep itself has been implicated in learning processes in both classes [15,23-25]. Despite the considerable differences in morphology and cytology of the mammalian neocortex and the avian telencephalon, recent gene expression profiling of the avian telencephalon during sleep and wakefulness have also identified a remarkable degree of overlap between vigilance state-related gene expression in these two vertebrate classes, suggesting that the cellular consequences of sleep and waking are phylogenetically conserved [26-28].

Notwithstanding these similarities, however, the available evidence for homeostatic sleep regulation in birds is conflicting. A declining trend in SWA across the night, a pattern similar to that observed in mammals, has been observed in the domestic hen [29], the European blackbird [30], and the white-crowned sparrow [25]. However, 24 hours of sleep deprivation in another avian species, the pigeon, resulted in small increases in the duration of total NREM and REM sleep in the subsequent sleep period, but no compensatory increase in SWA was reported [31]. Moreover, in the white-crowned sparrow during the migratory season, despite an average reduction in nocturnal sleep of 60%, no evidence of a compensatory increase in SWA was observed in the small amount of nocturnal sleep that was obtained [25]. Thus, whether birds have specialized mechanisms for dispensing with NREM sleep need remains unclear. The aim of the current study was therefore to ascertain whether sleep loss in the white-crowned sparrow leads to compensatory increases in SWA during the subsequent sleep period. To accomplish this goal, we investigated SWA dynamics in 6 wild-caught white-crowned sparrows, in baseline sleep relative to recovery sleep following 6 hours of sleep deprivation.

## Results

### Electrophysiological and behavioral correlates of baseline vigilance states

**Figure 1:**
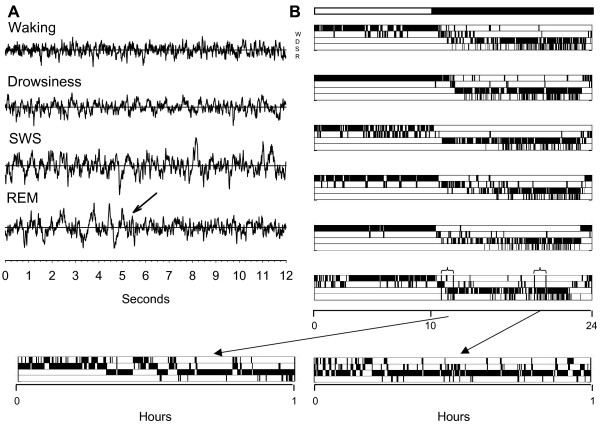
**EEG and behavioral criteria to define vigilance states in the white-crowned sparrow**. **A. **Representative examples of EEG recordings of each vigilance state. Arrow indicates onset of REM sleep. **B. **Hypnograms showing the 24-hour time course of waking (W), drowsiness (D), SWS sleep (S) and REM sleep (R) for each of the 6 birds during baseline. Behavioral states were scored in 4-sec epochs. However, because state transitions are frequent in sparrows, for each behavioral state only those episodes lasting > 16 seconds, and occurring without any interruption lasting > 16 seconds, are plotted. Bottom: two 1-hour periods with every 4-sec epoch plotted are shown to better illustrate the characteristic rapid transition between sleep to waking in sparrows.

Four behavioral states could be reliably distinguished based on visual inspection of the EEG and behavioral analysis: wakefulness, drowsiness, slow wave sleep (SWS) and REM sleep. Representative examples of EEG records of each vigilance state are shown in Figure 1A. Although movement artifacts were frequent during feeding and wing flapping, EEG recordings immediately before and after such behaviors showed a low-amplitude, high-frequency EEG in both hemispheres. As in other studies of songbirds [25,32], the EEG activity during drowsiness was intermediate between that of wakefulness and SWS, with increased amplitude in the low-frequency range relative to wakefulness. Behaviorally, birds in the drowsy state were readily distinguishable from wakeful birds or birds in SWS or REM sleep: they typically stood facing the center of the room with their head held close to the body and often moving from side to side. The position of the eyelids fluctuated between open, partially open, and completely closed. This behavior was intermittently interrupted by the birds opening their eyes completely, apparently in response to environmental stimuli. High-amplitude, low-frequency EEG activity reached its peak during SWS, when the birds were motionless, with eyes always closed and head either positioned beneath the wing, hanging forward or tucked in tightly toward the body. Sleep spindles, a characteristic feature of NREM sleep in mammals, were not visually detected, a finding consistent with other studies in birds [18]. Interhemispheric asymmetries in EEG during SWS were also not visually detected. REM sleep was characterized by low-amplitude high-frequency EEG activity similar to that occurring during wakefulness. REM sleep never occurred after an epoch of drowsiness, but only following an episode of unequivocal SWS. Since birds do not become atonic during REM sleep, activity recorded from the nuchal muscles is not always a reliable measure of this state in birds [18]. As a result, muscle recordings were not used in this study to distinguish behavioral states. However, behavioral signs of reduced muscle tone made REM sleep readily distinguishable from brief periods of awakening. Specifically, during REM sleep the eyes were closed and the head either rolled to one side or fell forward. Evidence of hypotonia was often indicated by drooping of the wing. In some cases, birds would sway and lose their balance on the perch. Figure 1B shows the 24-hour hypnograms during baseline for each of the 6 birds used in this study. The hypnograms on the bottom of this figure are representative examples of 1-hour recordings during the first and the last part of the dark phase, and show the rapid transition from one behavioral state to the other characteristic of sleep in these sparrows.

**Figure 2:**
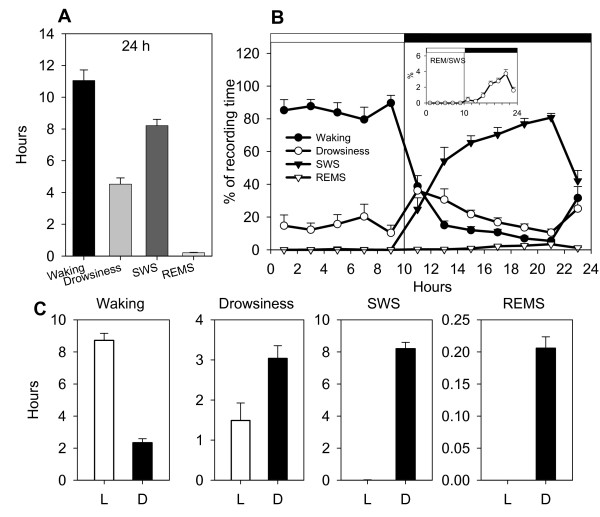
**Amount and time course of vigilance states during 24 hours of baseline**. **A. **Mean duration (± SEM, n = 6) of each vigilance state over the entire 24-hour baseline period. **B. **Time course of each vigilance state over the entire 24-hour baseline period. Values plotted as mean duration (± SEM, n = 6) of each state in 2-hour intervals. Inset shows the 24-hour time course of the ratio REM/SWS. **C**. Mean duration (± SEM, n = 6) of each vigilance state during the 10-hour light period (L) and the 14-hour dark period (D).

As previously described, white-crowned sparrows show a clear preference for diurnal waking and nocturnal sleep [25]. Specifically, as seen in Figure 1B and 2, SWS and REM sleep never occurred during the light phase, and while periods of drowsiness could be seen, their frequency varied across birds. Waking occupied 85.3 ± 4.3% of the light period and 17.0 ± 1.8% of the dark period, declining sharply following lights-off and reaching its minimum approximately 3 hours before lights-on (Fig. 2A–B). As described previously in other passerines [25,32], the percentage of drowsiness remained fairly constant throughout the 24-hour period, comprising 14.6 ± 4.3% of the light period and 22.0 ± 2.3% of the dark period. However, drowsiness showed a sharp increase in the first hour after lights-off, followed by a steady decline each hour until rising again in the hour before lights-on. SWS occupied most of the dark phase (59.5% ± 2.8). The first episode of SWS occurred approximately 1 hour after lights-off, and time spent in SWS rose steadily throughout the nocturnal period. State transitions during the nocturnal period were frequent, however, and SWS episodes were short, lasting on average only 5 minutes, interrupted by brief periods of drowsiness and wakefulness. REM occupied 1.5 ± 0.1% of the dark period (representing 1.8 ± 0.15% of total sleep time), a finding consistent with a number of published reports in both domestic and wild birds [21,29,31,33-36]. Although episodes of REM were short (mean ± SEM, 5.2 ± 0.2 sec), clusters of REM separated by a few seconds of SWS occurred frequently throughout the night. In a pattern similar to humans and other mammals [4,8] the proportion of time spent in REM sleep increased across the night, reaching a maximum 4 hours before lights-on (Fig. 2B, inset).

### Spectral analysis of baseline EEG

**Figure 3:**
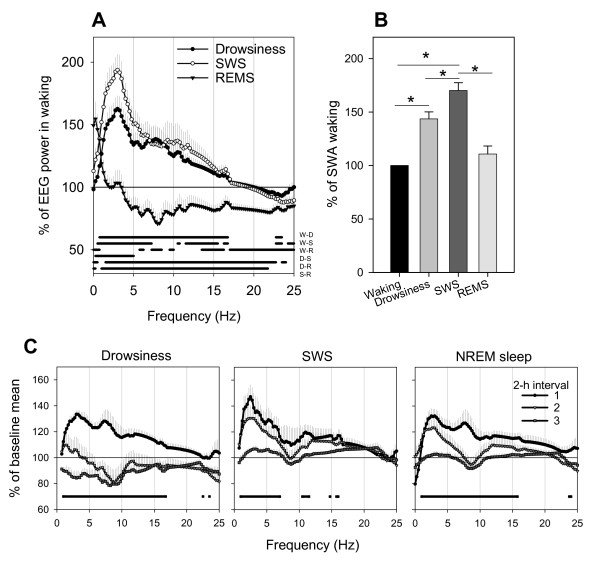
**Differences in the EEG power spectrum between vigilance states during the baseline dark period**. **A**. The relative EEG power spectrum of drowsiness, SWS, and REM sleep is plotted as percentage of the waking EEG power spectrum. Spectra refer to the first 6 hours of the dark period for all vigilance states except REM sleep, where the entire dark period contributed to the average spectrum. Values are means (± SEM, n = 6) of relative power density for each frequency bin (0.25 Hz, range: 0–25 Hz). The horizontal bars above the abscissa indicate frequency bins that differed significantly between vigilance states (p < 0.05, 2-tailed paired t-test). Waking (W), drowsiness (D), SWS sleep (S) and REM sleep (R). **B. **Mean slow wave activity (SWA, 0.5–4 Hz) in drowsiness, SWS and REM sleep expressed as percent of SWA in waking. Asterisks denote significant differences between states after correction for multiple comparisons.**C. **Time course of the EEG power spectrum in drowsiness and SWS and the two states combined (NREM) for the first three 2-hour intervals of the dark period. For each frequency bin (0.25 Hz) values are expressed as means (± SEM, n = 6) relative to the mean EEG power spectrum in that bin over the entire dark period in the corresponding vigilance state. Horizontal bar depicts frequency bins where 1-way repeated measures ANOVA (factor "time interval") was significant (p < 0.05)

Figure 3A shows the EEG power spectrum (0.5–25 Hz) during drowsiness, SWS and REM sleep expressed as percentage of the waking EEG spectrum. Relative to waking, both drowsiness and SWS showed significantly higher EEG power in the 0.5–15 Hz range, while REM sleep showed significant decreases for most frequencies > 13 Hz (Fig. 3A). The EEG power in the SWA range was significantly higher in SWS compared to waking, drowsiness, and REM sleep (Fig. 3B). SWA was also significantly higher in drowsiness relative to waking (Fig. 3B). As shown in Figure 3C, SWA declined across the dark period, being highest in the first 2-hour interval following lights-off. A similar trend of declining SWA was also evident for drowsiness, and when SWA was calculated for all of NREM sleep (Fig. 3C, right panel). Thus, SWA changes during baseline sleep mimic those seen in rodents and other mammals, i.e. SWA is highest at the beginning of the major sleep period and declines thereafter.

### Sleep deprivation and recovery

**Figure 4:**
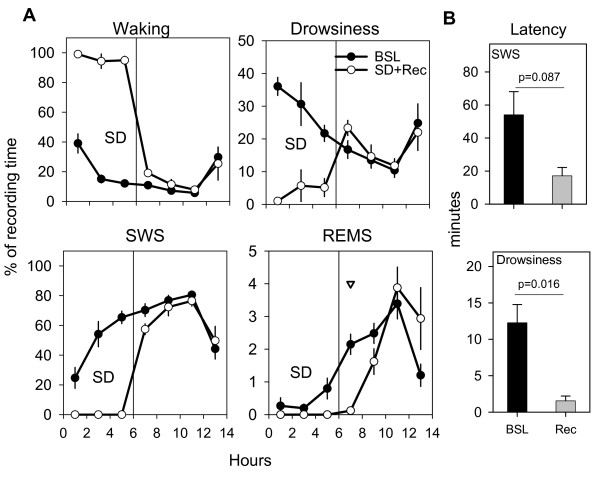
**Amount and time course of behavioral states during sleep deprivation and subsequent recovery**. **A. **Amount and time course of vigilance states during 6 hours of sleep deprivation (SD) and the first 8 hours of recovery sleep (Rec) and values during baseline (BSL). Values plotted as mean duration (± SEM, n = 6) of each state in 2-hour intervals expressed as percentage of total recording time. Triangle indicates a significant difference between the duration of vigilance state in BSL versus Rec. **B. **Latency to the first epoch scored as SWS or drowsiness during BSL and after SD. The latency to drowsiness was defined as the interval between lights-off during baseline or the end of 6 hours of SD and the first epoch of drowsiness. Values are plotted as mean (± SEM, n = 6) number of minutes from dark onset (BSL) or from the end of SD. Note the trend toward a decrease in SWS latency (p = 0.087, paired t-test) and the significant (p = 0.016, paired t-test) decrease in latency to drowsiness after SD.

Sparrows were subjected to total sleep deprivation during the first 6 hours of the dark period. The sleep deprivation procedure was effective at maintaining levels of wakefulness near 100% (96.1% ± 2.8). SWS and REM sleep were completely eliminated, although a small percentage of drowsiness did occur during the deprivation. The time course of vigilance states for the 6-hour sleep deprivation period followed by the 8-hour of recovery period is shown in Figure 4A. The amount and distribution of SWS and drowsiness did not differ during recovery relative to baseline, but the percentage of REM sleep decreased significantly during the first 2 hours of recovery. The latency to drowsiness decreased significantly after sleep deprivation, and the latency to SWS showed a trend toward a decrease (Fig. 4B).

**Figure 5:**
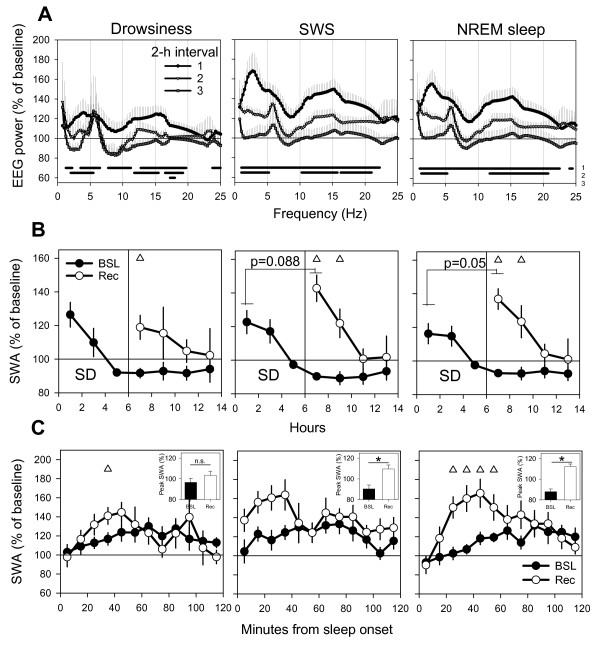
**EEG power spectrum and SWA during recovery sleep**. **A. **Time course of EEG power spectrum in drowsiness, SWS and the two states combined for the first three 2-hour intervals of recovery sleep. For each frequency bin (0.25 Hz) curves connect means (± SEM, n = 6) of relative power density expressed as percentage of mean values in that bin over the baseline dark period in the corresponding vigilance state. Horizontal bars depict frequency bins where the EEG power during recovery was significantly different from that during the corresponding baseline interval (p < 0.05, paired t-test). **B. **Time course of SWA (0.5–4 Hz) in drowsiness, SWS sleep and the two states combined. For each vigilance state, the 2-hour values are expressed as percentage of mean SWA during the baseline dark period in the corresponding vigilance state. Triangles depict significant difference between SWA during recovery sleep relative to the same circadian time during baseline sleep. Note that SWA in SWS and in NREM sleep (SWS + drowsiness) at the beginning of recovery sleep shows a trend to be higher (SWS, p = 0.08; NREM sleep, p = 0.05, paired t-test) than at the beginning of baseline sleep. **C**. Time course of SWA (0.5–4 Hz) in drowsiness, SWS sleep and the two states combined during the first 2 hours after sleep onset in baseline sleep and recovery sleep. For each vigilance state, values are expressed as percent of mean SWA during baseline for the corresponding vigilance state. Each data point represents mean (± SEM, n = 6) SWA over consecutive 10-min intervals. Triangles depict significant differences between SWA during recovery sleep relative to baseline sleep (p < 0.05, paired t-test). Inset depicts mean values (± SEM, n = 6) of peak SWA during the first 2 hours of baseline and recovery sleep analyzed over 30-min intervals. Asterisks indicate significant difference between baseline and recovery sleep (p < 0.05, paired t-test).

Figure 5A shows the changes for each 0.25 Hz frequency bin in the entire (0.5–25 Hz) EEG power spectrum for drowsiness, SWS, and the 2 states combined (NREM sleep) for the first three 2-hour intervals following sleep deprivation. During the first 2 hours of recovery, both drowsiness and SWS showed widespread increase in EEG power across most frequencies. These changes declined in the second 2-hour period, and disappeared almost completely by the sixth hour of recovery. Figure 5B shows changes for the SWA band (0.5–4.0 Hz) during the first 8 hours after sleep deprivation. Mean SWA during drowsiness was increased, relative to the same time of day in baseline, during the first 2 hours after sleep deprivation, but its levels in recovery were never higher than those reached at sleep onset during baseline. SWA during SWS was also higher, during the first 4 hours after sleep deprivation, relative to SWA at the same circadian time in baseline. Moreover, mean SWA during the first 2 hours of recovery was higher than the highest values reached in baseline after light offset, although the change did not reached statistical significance (p = 0.08). When SWA was calculated for drowsiness and SWS together, the mean 2-h value during recovery was increased at almost significance level (p = 0.05; Fig 5B). The relatively high variability in SWA changes after recovery was due to one single animal, which did not show appreciable SWA rebound after sleep deprivation.

Figure 5C shows the build-up of SWA during the first 2 hours after sleep onset at higher temporal resolution, i.e. by plotting SWA values in 10-min intervals. Relative to baseline, the SWA increase during recovery sleep was faster and reached significantly higher values in NREM sleep in the first hour after sleep onset. SWA in drowsiness also significantly increased in the first 20 to 40 minutes of recovery sleep relative to baseline. Finally, peak SWA in both SWS and NREM sleep during the first 2 hours after sleep onset reached significantly higher levels during recovery relative to baseline (p < 0.05).

**Figure 6:**
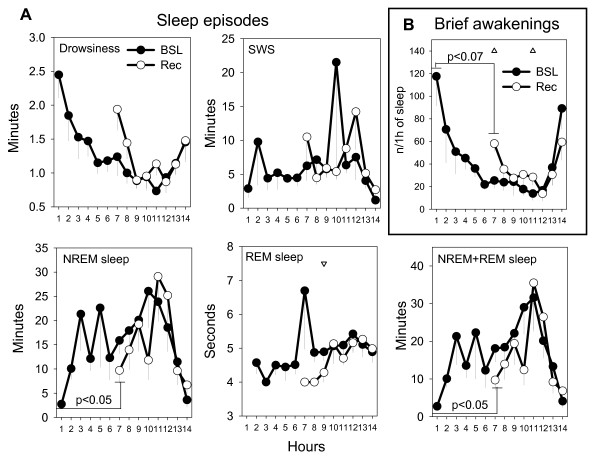
**Changes in sleep episodes duration and brief awakenings after sleep deprivation**. **A. **Duration of episodes of drowsiness, SWS, NREM, REM and total (NREM + REM) sleep during baseline (BSL) and recovery (Rec) sleep, plotted in 1-hour intervals beginning at sleep onset. Values (in minutes or seconds) are expressed as mean (± SEM, n = 6). For drowsiness, SWS and NREM an episode was defined as a period of continuous drowsiness or SWS lasting > 16 seconds and occurring without any waking interruption lasting > 16 seconds. A REM episode was defined as any occurrence of one or more consecutive epochs of REM sleep preceded and followed by any other behavioral state (no interruptions allowed). **B. **Number of brief awakenings during baseline (BSL) and recovery (Rec) sleep plotted in 1-hour intervals beginning at sleep onset. Values are expressed as mean (± SEM, n = 6) number of brief awakenings per hour of sleep. A brief awakening was defined as a brief waking period (<16 seconds) preceded and followed by at least one 4-sec epoch of SWS or drowsiness. Triangles depict significant difference between recovery sleep relative to the same circadian time during baseline sleep. Note that the number of brief awakenings at the beginning of recovery sleep shows a trend to be lower (p < 0.07, paired t-test) than at the beginning of baseline sleep.

In mammals, other markers of increased sleep intensity after sleep loss include an increase in the duration of sleep episodes, and a decrease in the number of brief awakenings [37-39]. Figure 6A shows the changes in sleep episode duration after sleep deprivation relative to baseline. During the first hour of recovery sleep, the episode duration for NREM sleep and total sleep (NREM + REM) increased significantly relative to the first hour of baseline sleep (p < 0.05), but not relative to the same time of day during baseline. The duration of REM sleep episodes did not differ at sleep onset after sleep deprivation relative to baseline, but it significantly decreased at hour 9 relative to the same time of day during baseline. Across the entire 8-hour recovery period (from the end of sleep deprivation until light onset) neither the mean duration of REM sleep episodes nor their number changed after sleep deprivation relative to baseline (mean ± SEM, baseline vs recovery, duration in seconds = 5.2 ± 0.2 vs 5.1 ± 0.2; number = 125.8 ± 4.0 vs 111.2 ± 17.5). Figure 6B shows that the number of brief awakenings showed a tendency to decrease (p < 0.07) during the first hour of recovery sleep relative to the first hour of baseline sleep. By contrast, the number of brief awakenings increased significantly at hour 7 and 11 in recovery sleep when compared to the same circadian time in baseline sleep.

## Discussion

The electrophysiological correlates of wakefulness and sleep we found in the white-crowned sparrow are similar to those described in other avian species [8]. The amount of REM sleep we report here, however, is lower than previously reported in the white-crowned sparrow [25]. One possible explanation for this discrepancy is the different criteria used to score REM sleep in the 2 studies. In the previous report vigilance state was sampled across the 24-hour period by scoring the first 4 seconds of each minute, whereas in the current study every 4-sec epoch was scored consecutively across the 24-hour period.

Interhemispheric EEG asymmetries have been described in a number of avian species [40], but we were unable to detect them in the white-crowned sparrow. This may reflect a genuine species difference, or may be due to technical limitations in our experimental setup. There are relatively small differences in the amplitude of the EEG signal between wakefulness and SWS in the bird, and visual scoring may fail to detect all but the most extreme examples of interhemispheric asymmetries. Another possible explanation is that unihemispheric sleep occurs mainly in some environmental conditions, for instance when birds are under the risk of predation [41,42], and may have little immediate adaptive value under standard housing conditions such as those used in our experiments.

The analysis of the relative power spectra in baseline sleep revealed that the EEG power in the SWA range (0.5–4 Hz) was significantly higher in NREM sleep relative to all other behavioral states, as expected. We also observed a marked decline in SWA over the course of the night, with EEG power in the 0.5–4 Hz range being highest in the first 2-h interval after sleep onset and declining steadily afterwards. During drowsiness, the EEG power in the SWA range was significantly higher relative to waking, and significantly lower relative to SWS, thus providing additional evidence that drowsiness can be considered a distinct state. Moreover, SWA in drowsiness also showed a declining trend across the nocturnal period similar to that seen in SWS, suggesting that in both states SWA is homeostatically regulated.

Following 6 hours of sleep deprivation, neither the amount nor the temporal distribution of SWS or drowsiness changed during the 12-hour recovery sleep period relative to baseline sleep. This result is consistent with the effects observed following short-term sleep deprivation in mammals, where often only markers of sleep intensity such as SWA change during recovery sleep, while NREM sleep duration may not increase. REM sleep duration also did not change during recovery sleep, but we observed a significant decrease in the latency to REM sleep in the first 2-hour interval of recovery sleep. In the rat, sleep deprivation of 3, 6 or 12 hours resulted in a small REM rebound [43,44], and in the mouse a prominent REM rebound was evident after deprivations as short as 6 hours [7]. In the pigeon, both 24 hours and long-term sleep deprivation (several days) resulted in a pronounced REM rebound, suggesting that REM is homeostatically regulated in the bird [31,45]. Thus, the lack of REM rebound in our study is likely a consequence of the short duration of the deprivation period, rather than a reflection of a lack of homeostatic regulation of REM sleep in this species.

The latency to the first episode of drowsiness decreased significantly during the recovery sleep period, and a trend toward a decrease in the latency to SWS was also observed. The decreased latency to drowsiness is perhaps not surprising given that drowsiness appears to represent a form of vigilant sleep and undoubtedly fulfills some of the requirements of sleep. Although sleep pressure may have been higher following the deprivation, the stress of having a predator (human) in the room for six hours may have induced a conflict between the need for continued vigilance and the need for sleep, and led to an immediate attempt to reduce sleep pressure through means other than frank sleep. Indeed, a previous report of migratory sleeplessness in the white-crowned sparrow indicated that although NREM sleep was reduced considerably during the migratory period, drowsiness was increased significantly, suggesting that drowsiness is capable of dissipating at least some level of sleep need [25].

Spectral analysis of the EEG following sleep deprivation also revealed changes in the SWA consistent with results described in mammals. Specifically, relative to baseline, SWA during recovery sleep reached significantly higher values in both SWS and NREM sleep in the first hour after sleep onset. SWA in drowsiness also significantly increased in the first 20 to 40 minutes after sleep onset. Finally, peak SWA in both SWS and NREM sleep during the first 2 hours after sleep onset reached significantly higher levels during recovery relative to baseline, indicating that at the beginning of baseline night SWA values were still below saturation. Interestingly, as in mammals, the first 2 hours of recovery sleep in sparrows were characterized by large increases in EEG power not only in the low frequency range, but also across most frequencies in the 10 to 20 Hz range. These changes in the high frequency range declined in the second 2-hour interval and disappeared entirely by the sixth hour of recovery sleep, and their origin and physiological significance remain unclear. It has recently been suggested, however, that prolonged wakefulness results in stronger cortico-cortical connections, which in turn would drive greater neuronal synchronization during sleep, leading to increased EEG power across all frequencies [17]. In sparrows, the increase in EEG power in the spindle frequency range (12–16 Hz) is especially intriguing, because spindles were not detected by visual scoring. In mammals, spindles and SWA reflect different but related aspects of cortical bistability, because they are thought to reflect the up states and the down states of the slow oscillation, respectively [46,47]. It is currently unknown whether this is also true in sparrows.

In mammals, sleep deprivation is followed not only by an increase in SWA, but also by an increase in the duration of sleep episodes and a decrease in the number of brief awakenings [37-39]. Human studies show that SWA during NREM sleep is mainly determined by the prior history of sleep and wakefulness and not by circadian mechanisms [48,49]. The evidence for the other markers of sleep consolidation is more limited, but suggests instead that both homeostatic and circadian factors are important [50]. As shown in Figure 6, when the first hour of recovery sleep was compared to the first hour of baseline sleep we found trends similar to those described in the mammalian literature (longer sleep episodes, fewer brief awakenings after sleep deprivation). However, when these behavioral measures were compared at the same time of day, changes were either no longer present (duration of NREM sleep episodes), or in the opposite direction (the number of brief awakenings increased after sleep deprivation). Thus, the current data show clear evidence for a post-sleep deprivation increase in sleep intensity as measured by SWA, while the evidence using other markers of sleep consolidation is mixed.

A previous report in the pigeon failed to identify increases in SWA following 24 hours of sleep deprivation [31], although a significant decline in waking duration following sleep deprivation was reported, suggesting that sleep pressure was indeed higher after the deprivation. As mentioned before, increased sleep pressure following deprivation may be discharged as an increase in sleep duration and/or intensity, and the duration of the deprivation may affect one parameter more than another. Thus, it is possible that the lack of a SWA rebound in the pigeon was due to the relatively long sleep deprivation procedure used in that study (24 hours). In rats, if wakefulness is enforced more than 12–24 hours, a significant amount of NREM may "leak" into the sleep deprivation period [51]. This raises the possibility that the pigeon was able to obtain significant amounts of SWA during the 24-hour deprivation, either through drowsiness or through local sleep within select neuronal populations. Furthermore, is worth noting that in our study the most significant effects of sleep loss on SWA were seen when we looked specifically at peak SWA or using high temporal resolution (10-min bins), which was not done in the pigeon study [31]. Further support for the idea of SWA leakage during prolonged sleep loss is provided by an early study of sleep deprivation using constant light in the pigeon [52]. The authors reported a near elimination of sleep for more than 10 days, with no subsequent increases in either total sleep time or SWA during recovery sleep. Based on these data, they concluded that pigeons do not show a mammalian-like compensatory rebound in SWA following sleep deprivation. However, the overall SWA was actually preserved across the entire sleep deprivation period in constant light, which may provide a reasonable explanation of why no rebound in SWA occurred during recovery sleep. Sleep restriction paradigms that preserve the overall 24-hour SWA amount are similarly not followed by SWA rebound [53]. Thus, SWA rebound is observed during recovery sleep after short but not after long sleep deprivation most likely because only the latter forces SWA to intrude into waking during the sleep deprivation period. In agreement with this interpretation, a recent report of recovery sleep in pigeons following short-term sleep deprivation (8 hours) shows that, like in sparrows, SWA does increase during recovery sleep relative to baseline sleep [54].

During the migratory season, captive white-crowned sparrows reduced nocturnal sleep by ~60%, with no evidence of increased SWA in the small amount of nocturnal sleep they do obtain [25]. Remarkably, despite this sleep loss, migrating birds remained capable of engaging in adaptive waking behaviors [25]. The preservation of performance and motivation during the migratory period suggests that migrants may have evolved adaptations to combat the negative consequences of sleep loss, or that natural selection may have promoted alternative mechanisms for sleep-loss compensation. Interestingly, during the migratory period, a significant increase in daytime drowsiness, as well as a trend toward an increase in nocturnal drowsiness was reported [25]. Although spectral analysis of nocturnal sleep was performed, no quantitative analysis of drowsiness was possible. The present findings suggest that short episodes of drowsiness are associated with a significant amount of EEG activity in the SWA range. Thus, it is possible that daytime drowsiness offers sufficient recuperative opportunities for the migratory white-crowned sparrow, while at the same time only marginally increasing the risk of predation. Interestingly, mammals that exhibit large amounts of drowsiness, such as ungulates, typically show reduced quantities of SWS, lending support to the idea that drowsiness can partially compensate for SWS [55]. A quantitative analysis of the EEG during migration will help address this issue definitively.

## Conclusion

The current study shows that in the white-crowned sparrow, as in all mammals studied to date, extending the period of wakefulness leads to compensatory increases in sleep intensity as measured by SWA during the subsequent sleep period. Since SWA homeostasis is thought to reflect sleep need and thus is a window on sleep function, these data may suggest that at least one fundamental function of sleep is conserved across species.

## Methods

### Animals

White-crowned sparrows were captured in their wintering grounds in the Sacramento valley in California (lat 39°00' N, long 122°00' E) in March 2005. All birds were collected using mist nets under the authority of a United States Fish and Wildlife Service permit. Birds were transported to the University of Wisconsin-Madison where they were individually housed in galvanized wire cages (L: 35 cm × W: 25 cm × H: 32 cm) in environmentally controlled rooms (40% relative humidity). Each bird was in visual and auditory contact with other birds in the room. The colony was exposed to photoperiodic conditions designed to simulate seasonal lighting changes. As a result of the fluctuating photoperiod, in the 14 days following surgery, birds were exposed to photoperiods ranging from 10:25; 13:75 light: dark (LD) to 10:00;14:00 LD. On the deprivation and recovery days light onset was at 07:30 and light offset at 17:30. Illuminance during the light phase was 560–640 lux measured at the level of the cage floor. Illuminance during the dark phase was less than 0.5 lux. Birds were fed a mixed-seed and provided water ad libitum, and their diet was supplemented daily with lettuce, dried insects, live mealworms, and grit. Six adult white-crowned sparrows were randomly selected from our captive population and surgically instrumented for chronic EEG recording. Since male and female white-crowned sparrows are not morphologically distinct, gender matching was not possible. Gender determination was made after death and shown to consist of 4 males and 2 females.

### EEG surgery

Surgical procedures were performed under isoflurane anesthesia (1.0%–3.5% isoflurane with 500 ml/min O2). The bird's head was stabilized in a stereotaxic device (Kopf Instruments, Tujunga, California, United States), cranial feathers were removed and an incision was made along the midline of the head to expose the cranium. Six small holes were drilled through the cranium to the dura: four holes for EEG electrodes, one for a reference electrode, and one for the ground electrode. Two holes were drilled on each hemisphere of the anterior forebrain 2 mm lateral to the midline over the hyperpallium (Wulst). Two additional holes were drilled 2 mm posterior to the anterior holes so that signal was recorded from both the anterior and posterior hyperpallium. Two holes were positioned over the midline of the cerebellum to accommodate the common reference electrode and the ground. Teflon insulated stainless steel electrodes (#791400, A-M Systems Sequim, Washington, United States) were inserted through the holes to the level of the dura and held in place with surgical glue. Each electrode was connected to a lightweight, flexible, and electrically shielded recording cable (Dragonfly, Ridgeley, West Virginia, United States). The cable was attached to the skull using dental acrylic (Justi Products, Oxnard, California, United States), and the incision was closed around the acrylic with surgical adhesive (Tissuemend II, Veterinary Products Laboratories, Phoenix, Arizona, United States). Following surgery, each bird was placed in the recording cage (L: 35 cm × W: 25 cm × H: 32 cm) for at least 14 days of postoperative recovery and adaptation to the recording cable. The recording cable was attached to a low-torque, six-channel mercury commutator (Dragonfly) designed for use with small birds. Under these conditions birds were able to move unimpeded throughout the cage.

### Sleep deprivation

Six birds were sleep deprived for six hours starting at dark onset. Throughout the sleep deprivation period, birds were never handled; walking quietly past the cages was sufficient to maintain vigilant wakefulness for the 6-hour period. To diminish the effects of stress, birds were acclimated to this procedure for 30 minutes every day during the week preceding the experimental day. In addition to electrophysiological recordings, behavior was also continuously recorded using 16 infrared-sensitive cameras (two per bird) connected to a digital video storage system (Salient Systems, Austin, Texas, United States). Infrared illuminators provided lighting for the cameras during the dark phase. Animal protocols followed the National Institutes of Health Guide for the Care and Use of Laboratory Animals and were in accordance with institutional guidelines.

### EEG acquisition and power spectrum analysis

EEG signals were obtained from two anterior and two posterior EEG channels for each bird during a 24-hour baseline day, a 6-hour sleep deprivation period starting at dark onset, and an 8-hour recovery period. Of the 4 available EEG derivations for each animal, the one with the smallest number of artifacts was selected for spectral analysis (left anterior n = 4, right anterior n = 1, left posterior n = 1). EEG signals were amplified, and band-pass filtered (0.1- to 30 Hz, additional notch filter was set at 60 Hz), using Grass-Telefactor amplifiers (model 12 Neurodata and 7P511, Grass-Telefactor, West Warwick, Rhode Island, United States), digitized at 1000 Hz, decimated to 100 Hz, and visualized using Somnologica 3 software (Medcare, Reykjavik, Iceland). EEG power spectra were computed for 4-sec epochs by a fast Fourier transform (FFT) routine (Hanning window, 0.25 Hz resolution) within the frequency range of 0.5–25.0 Hz. Differences were tested with ANOVA for repeated measures and two-tailed paired t-tests. For data and signal analysis the software package MATLAB (The Math Works, Inc., Natick, MA, USA) was used. Epochs that were scored but contained EEG artifacts were marked and omitted from further analysis of the spectra (number of artifacts during baseline, expressed as percentage of total 24-hour recording time: 46.5 ± 3.8% of recording time; more than 93% were waking artifacts). Differences in EEG spectra between baseline and recovery periods were tested with two-way ANOVA's for repeated measures. Contrasts were tested by post hoc two-tailed t-tests (for equal variances)

### Vigilance state scoring

Vigilance states were scored in 4-sec epochs using both EEG and behavioral recordings, and categorized as either wakefulness, drowsiness, SWS, or REM sleep. Because in birds nuchal muscle activity does not show a consistent or appreciable reduction in amplitude during REM sleep [29,31,32], we chose to rely on video monitoring of behavioral state to aid in the differentiation between REM and waking. Initial assessments of vigilance state were made based on visual inspection of the EEG as well as by analysis of recorded behavior using the following criteria. Wakefulness was characterized by a high-frequency low-amplitude EEG in both hemispheres. Behavior during wakefulness included hopping and flying around the cage, feeding, drinking, feather preening, and actively scanning the room. During drowsiness EEG activity was intermediate between that of wakefulness and SWS sleep (i.e., increased amplitude in the low-frequency range relative to wakefulness). During the drowsy state, birds held their heads close to their bodies, and the position of the eyelids fluctuated between open, partially closed, and completely closed states. During SWS sleep, EEG activity was dominated by slow waves of the highest amplitude. Behaviorally birds were motionless, with closed eyes; the head was either pulled in toward the body and facing forward or resting on the bird's back. A 4-sec epoch was scored as REM sleep if for > 2 sec the EEG amplitude was reduced by at least one-half the amplitude seen in the previous SWS episode and this amplitude reduction was accompanied by behavioral signs of REM including muscle hypotonia (feather or head drooping) or, rarely, eye movements.

## Abbreviations

D: drowsiness; EEG: electroencephalographic; FFT: fast Fourier transform; NREM sleep: non-rapid eye movement sleep; REM sleep: rapid eye movement sleep; SD: sleep deprivation; SWA: slow wave activity; SWS: slow wave sleep; W: wakefulness.

## Competing interests

The authors declare that they have no competing interests.

## Authors' contributions

SGJ carried out polygraphic recordings, EEG analysis, and wrote most of the manuscript; VVV performed EEG power spectrum analysis and generated all of the figures for the manuscript; RMB and GT coordinated the development of the study, provided the economical support to the project, and edited the last version of the manuscript; CC designed the experiments, coordinated and supervised the project, and helped draft the manuscript. All authors read and approved the final manuscript.

## References

[B1] Borbely AA (1982). A two process model of sleep regulation. Human Neurobiology.

[B2] Borbely AA, Tobler I, Hanagasioglu M (1984). Effect of sleep deprivation on sleep and EEG power spectra in the rat. Behav Brain Res.

[B3] Tobler I, Jaggi K (1987). Sleep and EEG spectra in the Syrian hamster (Mesocricetus auratus) under baseline conditions and following sleep deprivation. J Comp Physiol [A].

[B4] Achermann P, Dijk DJ, Brunner DP, Borbély AA (1993). A model of human sleep homeostasis based on EEG slow-wave activity: quantitative comparison of data and simulations. Brain Res Bull.

[B5] Dijk DJ, Daan S (1989). Sleep EEG spectral analysis in a diurnal rodent: Eutamias sibiricus. J Comp Physiol [A].

[B6] Tobler I, Franken P, Jaggi K (1993). Vigilance states, EEG spectra, and cortical temperature in the guinea pig. American Journal of Physiology.

[B7] Huber R, Deboer T, Tobler I (2000). Effects of sleep deprivation on sleep and sleep EEG in three mouse strains: empirical data and simulations. Brain Res.

[B8] Tobler I, M.H. Kryger TRWCD (2005). Phylogeny of sleep regulation. Principles and Practice of Sleep Medicine.

[B9] Vyazovskiy V, Borbely AA, Tobler I (2000). Unilateral vibrissae stimulation during waking induces interhemispheric EEG asymmetry during subsequent sleep in the rat. J Sleep Res.

[B10] Vyazovskiy VV, Welker E, Fritschy JM, Tobler I (2004). Regional pattern of metabolic activation is reflected in the sleep EEG after sleep deprivation combined with unilateral whisker stimulation in mice. Eur J Neurosci.

[B11] Vyazovskiy VV, Ruijgrok G, Deboer T, Tobler I (2006). Running wheel accessibility affects the regional electroencephalogram during sleep in mice. Cereb Cortex.

[B12] Schwierin B, Achermann P, Deboer T, Oleksenko A, Borbely AA, Tobler I (1999). Regional differences in the dynamics of the cortical EEG in the rat after sleep deprivation. Clin Neurophysiol.

[B13] Kattler H, Dijk DJ, Borbely AA (1994). Effect of unilateral somatosensory stimulation prior to sleep on the sleep EEG in humans. J Sleep Res.

[B14] Huber R, Deboer T, Tobler I (2000). Topography of EEG dynamics after sleep deprivation in mice. J Neurophysiol.

[B15] Huber R, Ghilardi MF, Massimini M, Tononi G (2004). Local sleep and learning. Nature.

[B16] Huber R, Ghilardi MF, Massimini M, Ferrarelli F, Riedner BA, Peterson MJ, Tononi G (2006). Arm immobilization causes cortical plastic changes and locally decreases sleep slow wave activity. Nat Neurosci.

[B17] Tononi G, Cirelli C (2006). Sleep function and synaptic homeostasis. Sleep Med Rev.

[B18] Rattenborg NC, Amlaner CJ, Lee-Chiong TL, Sateia MJ, Carskadon MA (2002). Phylogeny of sleep. Sleep medicine.

[B19] Amlaner CJ, Ball NJ, Kryger MH, Roth T, Dement WC (1994). Avian sleep. Principles and practice of sleep medicine.

[B20] Fuchs T, Siegel JJ, Burgdorf J, Bingman VP (2006). A selective serotonin reuptake inhibitor reduces REM sleep in the homing pigeon. Physiol Behav.

[B21] Szymczak JT (1987). Distribution of sleep and wakefulness in 24-h light-dark cycles in the juvenile and adult magpie, Pica pica. Chronobiologia.

[B22] Paredes SD, Terron MP, Cubero J, Valero V, Barriga C, Reiter RJ, Rodriguez AB (2006). Comparative study of the activity/rest rhythms in young and old ringdove (Streptopelia risoria): correlation with serum levels of melatonin and serotonin. Chronobiol Int.

[B23] Deregnaucourt S, Mitra PP, Feher O, Pytte C, Tchernichovski O (2005). How sleep affects the developmental learning of bird song. Nature.

[B24] Dave AS, Margoliash D (2000). Song replay during sleep and computational rules for sensorimotor vocal learning. Science.

[B25] Rattenborg NC, Mandt BH, Obermeyer WH, Winsauer PJ, Huber R, Wikelski M, Benca RM (2004). Migratory sleeplessness in the white-crowned sparrow (Zonotrichia leucophrys gambelii). PLoS Biol.

[B26] Cirelli C, Gutierrez CM, Tononi G (2004). Extensive and divergent effects of sleep and wakefulness on brain gene expression. Neuron.

[B27] Mackiewicz M, Shockley KR, Romer MA, Galante RJ, Zimmerman JE, Naidoo N, Baldwin DA, Jensen ST, Churchill GA, Pack AI (2007). Macromolecule biosynthesis - a key function of sleep. Physiol Genomics.

[B28] Jones S, Pfister-Genskow M, Benca RM, Cirelli C (2008). Molecular correlates of sleep and wakefulness in the brain of the white-crowned sparrow. J Neurochem.

[B29] van Luijtelaar EL, van der Grinten CP, Blokhuis HJ, Coenen AM (1987). Sleep in the domestic hen (Gallus domesticus). Physiol Behav.

[B30] Szymczak JT, Kaiser W, Helb HW, Beszczynska B (1996). A study of sleep in the European blackbird. Physiol Behav.

[B31] Tobler I, Borbély AA (1988). Sleep and EEG spectra in the pigeon (Columba livia) under baseline conditions and after sleep deprivation. Journal of Comparative Physiology A.

[B32] Szymczak JT, Helb HW, Kaiser W (1993). Electrophysiological and behavioral correlates of sleep in the blackbird (Turdus merula). Physiol Behav.

[B33] Ayala-Guerrero F, Vasconcelos-Duenas I (1988). Sleep in the dove Zenaida asiatica. Behav Neural Biol.

[B34] Ayala-Guerrero F, Mexicano G, Ramos JI (2003). Sleep characteristics in the turkey Meleagris gallopavo. Physiol Behav.

[B35] Szymczak JT (1986). Daily distribution of sleep states in the jackdaw, Corvus monedula. Chronobiologia.

[B36] Szymczak JT (1986). Sleep pattern in the rook, Corvus frugilegus. Acta Physiol Pol.

[B37] Franken P, Dijk DJ, Tobler I, Borbely AA (1991). Sleep deprivation in the rat: effects of electroencephalogram power spectra, vigilance states, and cortical temperature. American Journal of Physiology.

[B38] Trachsel L, Tobler I, Achermann P, Borbely AA (1991). Sleep continuity and the REM-nonREM cycle in the rat under baseline conditions and after sleep deprivation. Physiology and Behavior.

[B39] Tobler I, Franken P (1993). Sleep homeostasis in the guinea pig: similar response to sleep deprivation in the light and dark period. Neurosci Lett.

[B40] Rattenborg NC, Amlaner CJ, Lima SL (2000). Behavioral, neurophysiological and evolutionary perspectives on unihemispheric sleep. Neurosci Biobehav Rev.

[B41] Rattenborg NC, Lima SL, Amlaner CJ (1999). Half-awake to the risk of predation. Nature.

[B42] Rattenborg NC, Amlaner CJ, Lima SL (2000). Unihemispheric slow-wave sleep and predator detection in the pigeon (Columba livia). Sleep.

[B43] Tobler I, Borbély AA (1986). Sleep EEG in the rat as a function of prior waking. Electroenceph clin Neurophysiol.

[B44] Tobler I, Borbely AA (1990). The effect of 3-h and 6-h sleep deprivation on sleep and EEG spectra of the rat. Behavioral Brain Research.

[B45] Newman SM, Paletz EM, Rattenborg NC, Obermeyer WH, Benca RM (2008). Sleep deprivation in the pigeon using the Disk-Over-Water method. Physiol Behav.

[B46] Steriade M, Nunez A, Amzica F (1993). A novel slow (< 1 Hz) oscillation of neocortical neurons in vivo: depolarizing and hyperpolarizing components. J Neurosci.

[B47] Steriade M, Timofeev I, Grenier F (2001). Natural waking and sleep states, a view from inside neocortical neurons. Journal of Neurophysiology.

[B48] Dijk DJ, Beersma DG, Daan S (1987). EEG power density during nap sleep: reflection of an hourglass measuring the duration of prior wakefulness. Journal of Biological Rhythms.

[B49] Dijk DJ, Lockley SW (2002). Integration of human sleep-wake regulation and circadian rhythmicity. J Appl Physiol.

[B50] Akerstedt T, Hume K, Minors D, Waterhouse J (1998). Experimental separation of time of day and homeostatic influences on sleep. Am J Physiol.

[B51] Cirelli C, Tononi G, Kushida CA (2005). Total sleep deprivation. Sleep deprivation: basic science, physiology, and behavior.

[B52] Berger RJ, Phillips NH (1994). Constant light suppresses sleep and circadian rhythms in pigeons without consequent sleep rebound in darkness. Am J Physiol.

[B53] Kim Y, Laposky AD, Bergmann BM, Turek FW (2007). Repeated sleep restriction in rats leads to homeostatic and allostatic responses during recovery sleep. Proc Natl Acad Sci U S A.

[B54] Martinez-Gonzalez D, Lesku JA, Rattenborg NC (2008). Increased EEG spectral power density during sleep following short-term sleep deprivation in pigeons (Columba livia): evidence for avian sleep homeostasis. J Sleep Res.

[B55] Horne J (1988). Why We Sleep The Functions of Sleep in Humans and Other Mammals.

